# Association of Healthy Lifestyle with Insomnia Among Chinese Older Adults: A Cross-Sectional Study

**DOI:** 10.3390/clockssleep8020026

**Published:** 2026-05-09

**Authors:** Lu Liu, Wen Zhou, Yu Luo, Yueyi Zhang, Dongxi Wang, Ming Chen, Zhiming Wang, Yan Zeng

**Affiliations:** Brain Science and Advanced Technology Institute, Wuhan University of Science and Technology, Wuhan 430065, China; 18796520813@163.com (L.L.); 1532090194@wust.edu.cn (W.Z.); lylyly@wust.edu.cn (Y.L.); 15571724492@163.com (Y.Z.); 15137701676@163.com (D.W.); chen_ming2023@163.com (M.C.); 2418859272@wust.edu (Z.W.)

**Keywords:** healthy lifestyle, insomnia, older adults, healthy diet, cognitive activity

## Abstract

Insomnia is common among older adults and is associated with adverse health outcomes, yet evidence on its relationship with healthy lifestyle in Chinese older adults is limited. This study included 4929 participants from the Hubei Memory and Aging Cohort Study (HMACS). A healthy lifestyle score (range 0–6) was constructed based on body weight, drinking alcohol, smoking, regular exercise, diet, and cognitive activity. Participants were categorized into unfavorable (0–2), average (3), and favorable (4–6) lifestyle groups. Insomnia was defined using the Athens Insomnia Scale (AIS ≥ 6), or the Pittsburgh Sleep Quality Index (PSQI > 5). Multivariable logistic regression models were applied to assess the associations of overall and individual healthy behaviors with insomnia risk. Stratified analyses by smoking status and substitution analyses were conducted. Healthier lifestyle was associated with lower risk of insomnia. Compared with the unfavorable group, participants with favorable lifestyle had a 17.5% lower risk of insomnia. Among individual lifestyle behaviors, healthy diet and active cognitive activity were associated with reduced insomnia risk. Stratified analyses showed these associations were only evident among non-current smokers. Substitution analyses suggested that replacing unhealthy behaviors with healthy ones was associated with a lower insomnia risk. Favorable lifestyle, particularly healthy diet and active cognitive activity, is associated with lower insomnia risk among older adults, with stronger associations observed among non-current smokers.

## 1. Introduction

With the acceleration of global population aging, the number of older adults is increasing substantially. Currently, the global population aged 65 years and above is approximately 703 million, and it is projected to reach around 1.5 billion by 2050 [1]. This demographic shift has heightened attention to health issues in older adults, among which sleep health has emerged as a critical public health concern. Insomnia is one of the most prevalent sleep disorders in this population [2,3], with 30–60% of older adults reporting symptoms such as difficulty initiating sleep, frequent nighttime awakenings, and early morning awakenings [4,5]. Due to its chronic and persistent nature, untreated insomnia imposes substantial health burdens.

Extensive evidence shows that insomnia is closely associated with a range of adverse health outcomes. Compared with older adults with healthy sleep, those with long-term insomnia have significantly higher risks of depression [6], anxiety, cognitive decline [7,8], falls, reduced quality of life [9,10], and potentially even suicidal behaviors [11].

Behavioral therapy, particularly cognitive behavioral therapy for insomnia (CBT-I), has demonstrated sustained efficacy and is recommended as a first-line treatment for insomnia in older adults [12,13]. However, CBT-I primarily targets individuals who have already developed insomnia, and its widespread implementation is often constrained by limited access to trained providers, costs, and treatment adherence [14,15]. Meanwhile, hypnotic medications may increase the risk of falls, cognitive impairment, and dependence in older populations [16,17]. Therefore, identifying modifiable lifestyle factors before or at early stages of disease onset is crucial for reducing insomnia risk and promoting sleep health in older adults.

Unhealthy lifestyle behaviors such as poor diet [18], physical inactivity [19], smoking [20,21], and drinking alcohol [22,23] have been consistently linked to increased insomnia risk. However, most existing studies either focus on individual behaviors [19,24,25] or assess multi-dimensional lifestyle indices in general adult populations [22], and few have specifically examined older adults in China. Given that lifestyle behaviors tend to cluster, analyzing them in isolation may underestimate their combined impact on sleep health and limit the generalizability of findings.

To address this gap, this study, based on the Hubei Memory and Aging Cohort Study (HMACS), aimed to investigate the association between healthy lifestyle and insomnia risk among Chinese older adults. We constructed a composite index encompassing six modifiable lifestyle factors—body weight, drinking alcohol, smoking, regular exercise, diet, and cognitive activity—and assessed insomnia using the Athens Insomnia Scale (AIS) and Pittsburgh Sleep Quality Index (PSQI). The findings are expected to provide epidemiological evidence for the primary prevention of insomnia in this population.

## 2. Results

### 2.1. Demographic Characteristics

The baseline characteristics of the participants are summarized in Table 1. A total of 4929 participants were included in the analysis, of whom 2750 (55.8%) were women. The mean age was 72.61 ± 5.62 years, and 2660 participants (54.0%) had education above primary school. Most participants resided in rural areas (53.4%), were married (71.6%), and lived with family members (77.7%). Participants were categorized into three groups according to healthy lifestyle status: unfavorable, average, and favorable. Compared with the unfavorable group, participants in the favorable group were younger, more likely to be women, had higher educational attainment, and a lower prevalence of hypertension, cardiovascular disease, cerebrovascular disease, and insomnia (*p* < 0.001). Baseline characteristics of included and excluded participants are summarized in Supplementary Table S1. Most standardized mean differences (SMDs) were <0.25, indicating modest absolute differences between the two groups. Overall, the groups were broadly comparable, suggesting limited risk of selection bias.

### 2.2. Proportions of Healthy Lifestyle Behaviors in Different Groups

**Figure 1:**
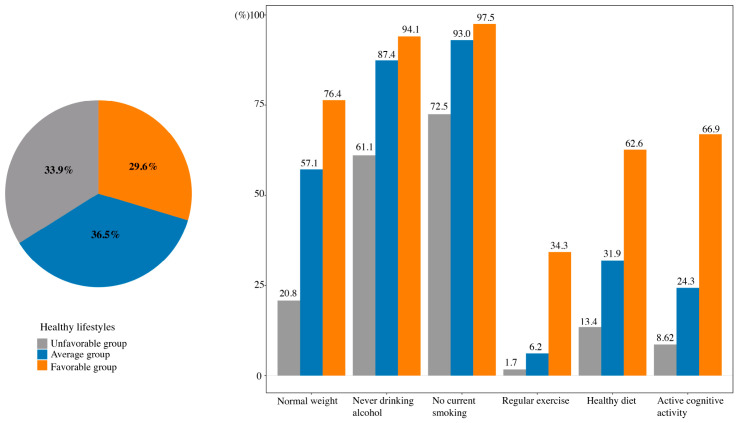
Proportions of each healthy lifestyle component in the unfavorable, average, and favorable lifestyle groups.

Among all participants, the prevalence of each healthy lifestyle behavior was as follows: normal body weight (50.5%), never drinking alcohol (80.4%), no current smoking (87.3%), regular exercise (12.9%), healthy diet (34.7%), and active cognitive activity (31.6%) (Supplementary Figure S1). Figure 1 shows the distribution of participants across lifestyle categories: 33.9% were classified as unfavorable, 36.5% as average, and 29.6% as favorable. By definition, participants in the favorable group had higher adherence to all healthy lifestyle behaviors compared with the average and unfavorable groups: normal body weight (76.4% vs. 57.1% vs. 20.8%; *p* < 0.001), never drinking alcohol (94.1% vs. 87.4% vs. 61.1%; *p* < 0.001), no current smoking (97.5% vs. 93.0% vs. 72.5%; *p* < 0.001), regular exercise (34.3% vs. 6.2% vs. 1.7%; *p* < 0.001), healthy diet (62.6% vs. 31.9% vs. 13.4%; *p* < 0.001), and active cognitive activity (66.9% vs. 24.3% vs. 8.6%; *p* < 0.001).

### 2.3. Association of Healthy Lifestyle with Insomnia

Table 2 presents the associations between healthy lifestyle and insomnia. In the unadjusted model (Model 1), participants in the favorable group had a significantly lower risk of insomnia compared with the unfavorable group (OR = 0.690, 95% CI: 0.596–0.798). This association remained after further adjustment for sociodemographic factors and medical history. In the fully adjusted model (Model 3), participants in the favorable group had a 17.5% lower risk of insomnia compared with the unfavorable group (OR = 0.825, 95% CI: 0.702–0.968), whereas the difference between the average and unfavorable groups was not statistically significant (OR = 0.972, 95% CI: 0.845–1.117). Trend analysis indicated a significant inverse association between healthy lifestyle level and insomnia risk (*p* for trend = 0.024).

### 2.4. Association of Healthy Lifestyle Behaviors with Insomnia

After adjustment, healthy diet (OR = 0.762, 95% CI: 0.669–0.866) and active cognitive activity (OR = 0.732, 95% CI: 0.634–0.844) were associated with lower risk of insomnia, suggesting potential protective effects. In contrast, normal body weight (OR = 0.949, 95% CI: 0.842–1.070), regular exercise (OR = 1.104, 95% CI: 0.919–1.326), and never drinking alcohol (OR = 1.044, 95% CI: 0.891–1.225) were not significantly associated with insomnia risk. Notably, non-current smokers had a higher risk of insomnia compared with current smokers (OR = 1.386, 95% CI: 1.142–1.687) (Table 3). Neither interaction term reached statistical significance (Supplementary Table S2).

### 2.5. Associations Between Healthy Lifestyle and Insomnia Stratified by Smoking Status

Given the higher insomnia risk among non-current smokers compared with current smokers, stratified analyses by smoking status were conducted. Among non-current smokers, those in the favorable group had a significantly lower risk of insomnia compared with the unfavorable group in the fully adjusted model (OR = 0.738, 95% CI: 0.620–0.878), whereas the association for the average group, although in the same direction, was not statistically significant (OR = 0.903, 95% CI: 0.774–1.052). Trend analysis indicated a significant inverse association between healthy lifestyle level and insomnia risk (*p* for trend < 0.001). Among current smokers, the association between healthy lifestyle and insomnia followed a similar direction but was not statistically significant (*p* for trend = 0.105) (Table 4).

### 2.6. Substitution Analysis of Healthy Lifestyle Behaviors Stratified by Smoking Status

**Figure 2:**
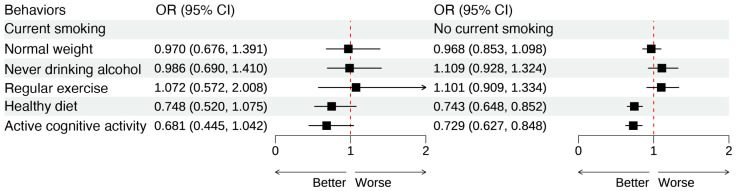
Substitution analysis of healthy lifestyle behaviors and insomnia risk stratified by smoking status; OR represents the change in insomnia risk associated with replacing an unhealthy behavior with a healthy one, while holding the overall healthy lifestyle score of other behaviors constant. Model adjusted for age, sex, residence, marital status, education, living arrangement, hypertension, diabetes, hyperlipidemia, cardiovascular disease, and cerebrovascular disease. Each black square represents the OR, and the horizontal line shows the 95% CI.

In the substitution analysis, each healthy lifestyle behavior was treated as a binary variable (0 = unhealthy, 1 = healthy), and the substitution effect was examined while keeping the total score of other lifestyle behaviors constant. Among non-current smokers, healthy diet (OR = 0.743, 95% CI: 0.648–0.852) and active cognitive activity (OR = 0.729, 95% CI: 0.627–0.848) were significantly associated with lower risk of insomnia. In contrast, among current smokers, none of the substitution effects reached statistical significance (*p* > 0.05). These findings suggest that replacing unhealthy behaviors with healthy ones may reduce the risk of insomnia, particularly among non-current smokers (Figure 2 and Supplementary Table S3).

### 2.7. Sensitivity Analyses

**Figure 3:**
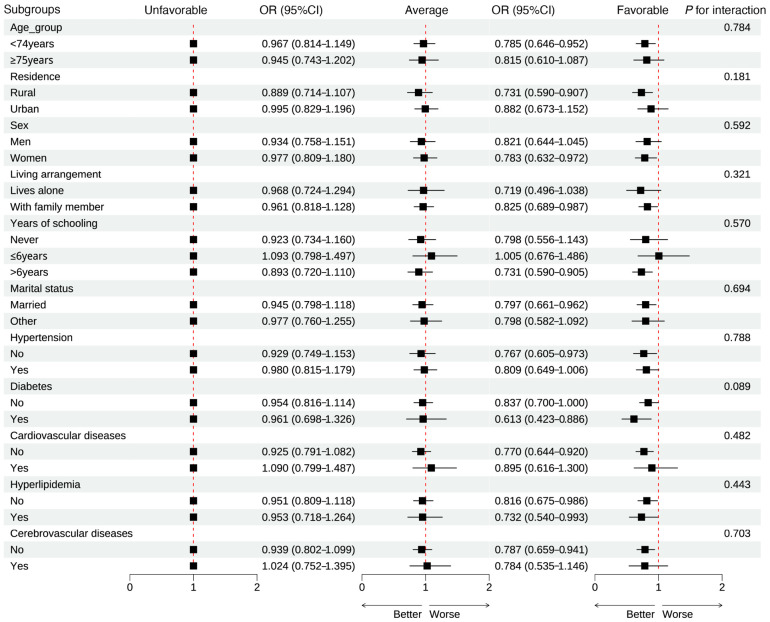
Association of healthy lifestyle and the risk of insomnia stratified by covariates. Model adjusted for age, sex, residence, marital status, education, living arrangement, hypertension, diabetes, hyperlipidemia, cardiovascular disease, and cerebrovascular disease. Each black square represents the OR, and the horizontal line shows the 95% CI.

Subgroup analyses showed that the association between healthy lifestyle and insomnia risk was generally consistent across subgroups defined by age, sex, residence, living arrangement, education, marital status, and the presence of hypertension, diabetes, cardiovascular disease, hyperlipidemia, or cerebrovascular disease. Compared with the unfavorable group, participants in the favorable group had lower insomnia risk in most subgroups, with no significant interactions observed (all *p* for interaction > 0.05) (Figure 3). Among PSQI-defined insomnia cases, using a symptom-based definition (difficulty initiating sleep, difficulty maintaining sleep, or early-morning awakening) yielded similar associations with healthy lifestyle (Supplementary Table S4).

Stratification by smoking status indicated that among non-current smokers, higher levels of healthy lifestyle were significantly associated with lower insomnia risk, whereas among current smokers, the association was in the same direction but not statistically significant (Supplementary Table S5). We separated the analyses based on available AIS and PSQI data, both of which were used separately to assess insomnia. While AIS data showed no significant association, PSQI data revealed that a favorable lifestyle was associated with a lower risk of insomnia (Supplementary Tables S6 and S7). The association between healthy lifestyle and insomnia risk stratified by smoking status (never, former, or current smokers) is shown in Supplementary Table S8. For never-smokers, a favorable lifestyle was significantly associated with a lower risk of insomnia. Analyses using unimputed datasets yielded results that were generally consistent with the primary analyses. Although the overall association between a favorable lifestyle and insomnia did not reach statistical significance, stratified analyses indicated a significantly lower risk of insomnia among non-current smokers (OR = 0.744, 95% CI: 0.617–0.896), whereas no significant association was observed among current smokers (Supplementary Tables S9 and S10). When lifestyle scores were treated as a continuous variable, higher scores were linearly associated with a lower risk of insomnia. (Supplementary Table S11 and Figure S2). E-value analysis indicated that an unmeasured confounder would need to be associated with both a favorable healthy lifestyle and insomnia by at least 1.72-fold to fully explain the observed OR of 0.825 (lower CI limit 0.702, E-value 1.22), suggesting reasonable robustness to unmeasured confounding.

## 3. Discussion

Based on cross-sectional data from the HMACS, this study primarily evaluated the association between overall healthy lifestyle and insomnia risk among Chinese older adults. Our main finding indicated that a favorable overall lifestyle was significantly associated with a lower risk of insomnia. In addition, exploratory analyses of individual lifestyle behaviors suggested that healthy diet and active cognitive activity showed the most consistent inverse associations with insomnia, whereas body weight, regular exercise, and alcohol consumption were not significantly associated. Notably, the association between overall healthy lifestyle and insomnia appeared to vary by smoking status, although further studies are needed to confirm these findings.

Current evidence regarding the relationship between healthy lifestyle and insomnia among Chinese older adults remains limited [22]. Overall, our findings are consistent with previous studies linking healthy lifestyle to better sleep health [26,27,28], indicating that favorable lifestyle is associated with a lower risk of insomnia. A prior cross-sectional study among Chinese adults showed that healthier lifestyles were consistently associated with better overall sleep health [22]. The present study extends these findings to an older adult population, providing additional population-specific evidence for the potential protective role of healthy lifestyle in sleep health.

Among individual lifestyle behaviors, healthy diet may contribute to better sleep through several biological pathways, including modulation of neurotransmitters and melatonin secretion [18,29], reduction in chronic inflammation and oxidative stress [30,31], and the improvement of hypothalamic–pituitary–adrenal axis function and neurotrophic factor levels [32,33]. Active cognitive activity may maintain neuronal connectivity [34,35], and cognitive function [36] and promote sleep homeostasis. These activities are often accompanied by social participation, which provides social support, encourages other healthy behaviors, and may alleviate loneliness [37,38]. However, the relationship between cognitive activity and sleep may be bidirectional; individuals with better cognitive function may be more likely to engage in cognitive activities, which in turn may further promote sleep quality. Longitudinal studies are warranted to clarify the directionality of this relationship. In contrast, no significant associations were observed between body weight, regular exercise, or drinking alcohol and insomnia. Several factors may explain these findings. First, body mass index (BMI) may not reflect body fat distribution, sarcopenia, or metabolic health, which could be more directly related to sleep [39,40]. Second, although physical activity was defined according to guidelines, the physiological response to exercise in older adults may be attenuated by medications or psychological factors [41,42]. Moreover, the cross-sectional design may not capture long-term cumulative effects of regular exercise on sleep. Finally, the overall prevalence and level of alcohol consumption in this population were low, and drinking patterns and amounts were not further differentiated, which may have limited the ability to detect potential effects of alcohol on insomnia risk.

Notably, the association between smoking and insomnia risk does not fully align with conventional expectations [43,44]. Although current smokers appeared to have a lower risk of insomnia compared with non-current smokers, this finding should not be interpreted as a protective effect of smoking on sleep. Rather, it may reflect non-causal mechanisms, such as withdrawal-related symptom relief (e.g., reduced anxiety or irritability), sleep disturbances among recent quitters, residual confounding, or reverse causation [45,46,47]. Further stratified and substitution analyses indicated that the inverse association between healthy lifestyle and insomnia risk was primarily observed among non-current smokers, whereas associations among current smokers were weaker and not statistically significant. These findings indicate that the beneficial association of healthy lifestyle with sleep health appears more evident among non-current smokers. Several mechanisms may contribute to the observed differences by smoking status. Nicotine may disrupt sleep through alterations in neurotransmitter balance and circadian rhythm regulation [48], as well as by imposing physiological burden related to chronic inflammation and cardiovascular risk [49,50], which could reduce the potential benefits of healthy behaviors.

In addition, current smokers often experience higher stress levels, poorer psychological well-being, and lower social support [51,52], which may further weaken the effect of a healthy lifestyle on sleep. Previous studies also suggest that sleep impairment may be more strongly associated with nicotine dependence severity rather than smoking status [53], which may partly explain the variation in associations observed across smoking groups. The substitution analysis further indicated that replacing unhealthy behaviors with healthy diet or active cognitive activity was associated with a significantly lower risk of insomnia among non-current smokers, whereas such substitution effects were not statistically significant among current smokers. These findings highlight the potential benefits of overall lifestyle improvement for sleep health and suggest that lifestyle-based interventions may be more effective among non-smoking older adults.

This study has several strengths. First, it was based on a large-scale older adult population from the HMACS, systematically evaluating the associations between healthy lifestyle and its individual behaviors and insomnia, providing important epidemiological evidence for Chinese older adults. Second, the study not only examined overall healthy lifestyle, but also assessed the independent effects of each behavior and their differences across smoking status. Furthermore, by incorporating substitution analysis, we explored the potential impact of replacing unhealthy behaviors with healthy ones on insomnia risk, thereby enriching the scientific basis for lifestyle interventions.

However, several limitations should be acknowledged. First, the cross-sectional design limits causal inference and cannot exclude reverse causation or residual confounding. Second, healthy behaviors were primarily self-reported, which may introduce information bias. Third, although correlations, multicollinearity checks, and multiple correspondence analysis (MCA) supported the independence of the variables, potential clustering of lifestyle behaviors cannot be entirely excluded; future studies could apply structural equation modeling to further explore the complex pathways among these behaviors. In addition, objective sleep assessments, including polysomnography, were not available in the baseline survey; therefore, comorbid sleep-related breathing disorders such as obstructive sleep apnea could not be evaluated or excluded, which may have contributed to some misclassification of insomnia status. Future longitudinal studies or intervention trials are warranted to confirm the causal effects and elucidate the underlying mechanisms linking healthy lifestyle behaviors to insomnia in older adults.

## 4. Methods

### 4.1. Study Design and Participants

This cross-sectional study was based on the HMACS, officially registered in 2018 (ChiCTR1800019164). The HMACS recruited older adults aged 60 years and above from Hubei Province, China. Both baseline and follow-up assessments collected information on participants’ sociodemographic characteristics, clinical measures, lifestyle, sleep-related information, chronic disease history, and comprehensive cognitive function. Detailed descriptions of the study design, participant recruitment, and data collection methods have been reported previously [54,55]. All participants provided written informed consent prior to participation.

**Figure 4:**
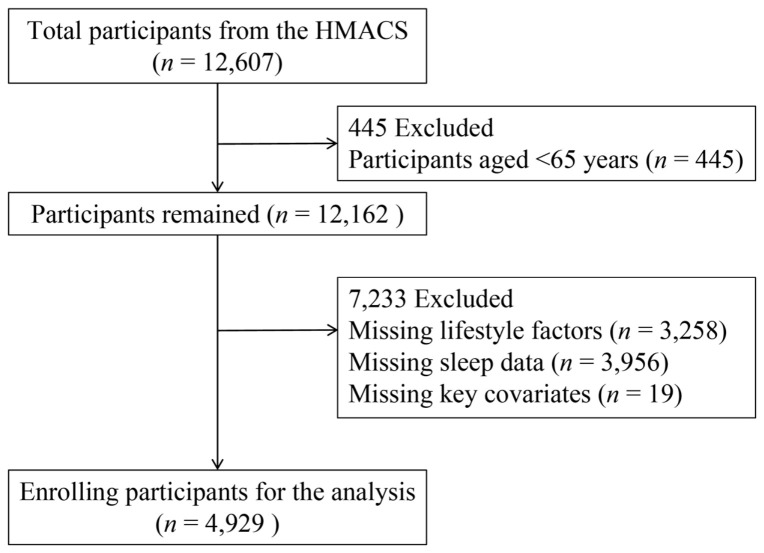
The flowchart of study design.

Between May 2018 and July 2025, a total of 12,607 participants completed the health screening questionnaire. For this study, we included 12,162 older adults aged 65 years and above, sequentially excluding those with missing data on lifestyle factors (*n* = 3258), sleep information (*n* = 3956), or key covariates (*n* = 19). Ultimately, 4929 participants were included in the final analysis (Figure 4).

### 4.2. Assessment of Healthy Lifestyle

Participants’ lifestyle was assessed based on six modifiable lifestyle factors: body weight, drinking alcohol, smoking, regular exercise, diet, and cognitive activity. Body weight was considered healthy if BMI ranged from 18.5 to 24.9 kg/m^2^ [22,56]. Regarding drinking alcohol and smoking, never drinking and no current smoking were defined as healthy behaviors [57,58]. Regular exercise was assessed by recording participants’ weekly frequency and total duration; engaging in at least 150 min of moderate-intensity or 75 min of vigorous-intensity exercise per week was considered healthy [59,60]. Dietary assessment included six food components (vegetables, fruits, red meat, fish, tea, and coffee), with participants in the top 40% of intake frequency defined as having a healthy diet [33]. Cognitive activity, including reading, playing chess or cards, calligraphy, and painting, was considered healthy if performed at least three times per week [61].

Each factor meeting the healthy criterion was assigned 1 point, with 0 points otherwise. The sum of all six factors constituted the healthy lifestyle score (range 0–6), with higher scores indicating better adherence to a healthy lifestyle. Based on the sample distribution, participants were categorized into three groups: favorable (scores 4–6), average (score 3), and unfavorable (scores 0–2).

### 4.3. Assessment of Insomnia

Insomnia was assessed using the AIS and the PSQI. The AIS is a validated, 8-item scale based on the International Classification of Diseases, 10th Edition, with each item scored from 0 (no problem) to 3 (severe problem) [62]. The total score ranges from 0 to 24, with higher scores indicating more severe insomnia. The PSQI comprises 19 items generating seven component scores, with a global score ranging from 0 to 21; higher scores denote poorer sleep quality [63]. Both the AIS and PSQI have been widely used and validated in clinical and research settings for insomnia screening, showing high diagnostic accuracy, reliability, and feasibility for use in large populations [64].In this study, insomnia was primarily defined as an AIS score ≥ 6 or a PSQI score > 5 [63,65,66]. In sensitivity analyses, among participants identified as having insomnia based on the PSQI criterion, insomnia was alternatively defined according to the presence of difficulty initiating sleep, difficulty maintaining sleep, or early-morning awakening with inability to return to sleep [3]. All sleep assessments were conducted by trained medical researchers following standardized procedures. However, the HMACS baseline survey did not include polysomnography or physician-diagnosed sleep apnea assessment.

### 4.4. Covariates

Covariates included sociodemographic factors and medical history. Sociodemographic factors included age, sex, residence, years of education, marital status, and living arrangement. Medical history included self-reported physician-diagnosed hypertension, diabetes, hyperlipidemia, cardiovascular disease, and cerebrovascular disease. Missing data were addressed using five imputed datasets generated with the mice package in R. Models were fitted in each dataset and combined using Rubin’s rules. The proportion of missing data for each covariate is summarized in Supplementary Table S12.

### 4.5. Statistical Analysis

Continuous variables were presented as means ± SD, and categorical variables as frequencies (percentages). Group differences were assessed using independent *t*-tests or ANOVA for normally distributed continuous variables, Kruskal–Wallis tests for non-normally distributed variables, and Chi-square tests for categorical variables. Prior to regression analyses, correlations among individual lifestyle behaviors were examined (Supplementary Figure S3). Multicollinearity among lifestyle behaviors and covariates was assessed using variance inflation factors (VIFs) (Supplementary Table S13). In addition, MCA was conducted to evaluate potential clustering patterns among the six lifestyle behaviors, and no clear aggregation was observed (Supplementary Table S14).

Logistic regression models were used to examine the associations between healthy lifestyle (overall and individual behaviors) and insomnia risk, with results reported as odds ratios (ORs) and 95% confidence intervals (CIs). Three models were constructed: Model 1, unadjusted; Model 2, adjusted for age, sex, residence, marital status, education, and living arrangement; Model 3, further adjusted for hypertension, diabetes, hyperlipidemia, cardiovascular disease, and cerebrovascular disease based on Model 2. Potential interactions between lifestyle behaviors were assessed by including two prespecified multiplicative interaction terms (healthy diet × active cognitive activity and regular exercise × non-current smoking) in the regression models.

Stratified analyses were performed by smoking status. Logistic regression models were applied to evaluate the associations between healthy lifestyle (excluding the smoking component) and insomnia risk. In addition, substitution models were performed separately among current and non-current smokers to estimate the potential effects of replacing unhealthy lifestyle behaviors with healthy ones on insomnia risk, while holding the total score of other lifestyle behaviors constant. To test the robustness of the findings, several sensitivity analyses were conducted: (1) subgroup analyses stratified by covariates (e.g., sex, age, and chronic disease status); (2) analyses restricted to participants with available AIS and PSQI scores for insomnia assessment; (3) analyses applying an alternative symptom-based definition of insomnia rather than the PSQI cut-off, where insomnia was defined as difficulty initiating sleep, difficulty maintaining sleep, and early-morning awakening with inability to return to sleep; (4) stratified analyses using three-category smoking status (never, former, and current); (5) repetition of the primary analyses using unimputed data; (6) examination of the association between lifestyle scores and insomnia using linear regression to assess linear relationships, and restricted cubic splines analyses to evaluate potential non-linear associations; and (7) sensitivity to potential unmeasured confounding (e.g., OSA or psychological factors), evaluated using E-values. All statistical analyses were performed using RStudio (version 4.3.3). All *p*-values were two-sided, and *p* < 0.05 was considered significant.

## 5. Conclusions

The findings of this study indicate that a favorable healthy lifestyle is significantly associated with a lower risk of insomnia among Chinese older adults, with healthy diet and active cognitive activity showing the most consistent protective effects. These associations were primarily observed among non-current smokers, suggesting that the relationship between healthy lifestyle and sleep health may vary by smoking status. Future studies using longitudinal or interventional designs are warranted to clarify the causal relationships and underlying mechanisms, providing evidence for targeted sleep health interventions.

## Figures and Tables

**Table 1:** Baseline characteristics of study population.

	Overall (*n* = 4929)	Unfavorable (*n* = 1671)	Average (*n* = 1799)	Favorable (*n* = 1459)	*p*-Value
Age (mean (SD))	72.61 (5.62)	72.90 (5.52)	72.77 (5.64)	72.07 (5.67)	<0.001
Sex (%)					<0.001
Men	2179 (44.2)	810 (48.5)	764 (42.5)	605 (41.5)	
Women	2750 (55.8)	861 (51.5)	1035 (57.5)	854 (58.5)	
Residence (%)					<0.001
Urban	2298 (46.6)	1100 (65.8)	900 (50.0)	298 (20.4)	
Rural	2631 (53.4)	571 (34.2)	899 (50.0)	1161 (79.6)	
Marital status (%)					<0.001
Married	3485 (71.6)	1110 (67.3)	1252 (70.3)	1123 (78.2)	
Other	1383 (28.4)	540 (32.7)	530 (29.7)	313 (21.8)	
Years of schooling (mean (SD))	6.85 (5.35)	5.11 (4.97)	6.22 (5.36)	9.61 (4.65)	<0.001
Years of schooling (%)					<0.001
Never	1388 (28.2)	625 (37.4)	605 (33.6)	158 (10.8)	
≤6 years	881 (17.9)	407 (24.4)	313 (17.4)	161 (11.0)	
>6 years	2660 (54.0)	639 (38.2)	881 (49.0)	1140 (78.1)	
Living arrangement (%)					<0.001
Lives alone	1100 (22.3)	434 (26.0)	407 (22.6)	259 (17.8)	
With a family member	3829 (77.7)	1237 (74.0)	1392 (77.4)	1200 (82.2)	
Smoking (%)					<0.001
Never	3819 (77.5)	1000 (59.8)	1514 (84.2)	1305 (89.4)	
Former	488 (9.9)	211 (12.6)	159 (8.8)	118 (8.1)	
Current	622 (12.6)	460 (27.5)	126 (7.0)	36 (2.5)	
Drinking alcohol (%)					<0.001
Never	3967 (80.5)	1021 (61.1)	1573 (87.4)	1373 (94.1)	
Former	381 (7.7)	251 (15.0)	94 (5.2)	36 (2.5)	
Current	581 (11.8)	399 (23.9)	132 (7.3)	50 (3.4)	
Regular exercise (%)	639 (13.0)	28 (1.7)	111 (6.2)	500 (34.3)	<0.001
Healthy diet (%)	1712 (34.7)	224 (13.4)	574 (31.9)	914 (62.6)	<0.001
Active cognitive activity (%)	1558 (31.6)	144 (8.6)	438 (24.3)	976 (66.9)	<0.001
BMI (mean (SD))	23.70 (3.52)	24.70 (3.90)	23.51 (3.50)	22.80 (2.73)	<0.001
BMI (%)					<0.001
Underweight	266 (5.4)	176 (10.5)	66 (3.7)	24 (1.6)	
Normal	2490 (50.5)	347 (20.8)	1028 (57.1)	1115 (76.4)	
Overweight	1651 (33.5)	883 (52.8)	518 (28.8)	250 (17.1)	
Obesity	522 (10.6)	265 (15.9)	187 (10.4)	70 (4.8)	
Diabetes (%)	928 (18.8)	310 (18.6)	347 (19.3)	271 (18.6)	0.821
Hypertension (%)	2672 (54.2)	979 (58.6)	1002 (55.7)	691 (47.4)	<0.001
Hyperlipidemia (%)	1267 (25.7)	388 (23.2)	443 (24.6)	436 (29.9)	<0.001
Cardiovascular disease (%)	913 (18.5)	331 (19.8)	357 (19.8)	225 (15.4)	0.001
Cerebrovascular disease (%)	896 (18.2)	369 (22.1)	326 (18.1)	201 (13.8)	<0.001
Insomnia (%)	1948 (39.5)	713 (42.7)	740 (41.1)	495 (33.9)	<0.001

SD: Standard Deviation.

**Table 2:** Associations between healthy lifestyle and insomnia risk.

Healthy Lifestyle	Model 1		Model 2		Model 3	
	OR (95% CI)	*p*	OR (95% CI)	*p*	OR (95% CI)	*p*
Unfavorable	ref		ref		ref	
Average	0.939 (0.820, 1.075)	0.360	0.959 (0.835, 1.102)	0.556	0.972 (0.845, 1.117)	0.686
Favorable	0.690 (0.596, 0.798)	<0.001	0.800 (0.682, 0.937)	0.006	0.825 (0.702, 0.968)	0.019
*p* for trend	0.835 (0.777, 0.897)	<0.001	0.898 (0.830, 0.972)	0.008	0.912 (0.842, 0.988)	0.024

Model 1: unadjusted; Model 2: adjusted for age, sex, residence, marital status, education, and living arrangement; Model 3: adjusted for age, sex, residence, marital status, education, living arrangement, hypertension, diabetes, hyperlipidemia, cardiovascular disease, and cerebrovascular disease.

**Table 3:** Association of each healthy lifestyle behavior with insomnia risk.

Healthy Lifestyle Behaviors	Model 1		Model 2		Model 3	
	OR (95% CI)	*p*	OR (95% CI)	*p*	OR (95% CI)	*p*
Normal weight	0.925 (0.825, 1.036)	0.179	0.933 (0.830, 1.048)	0.241	0.949 (0.842, 1.070)	0.394
Never drinking alcohol	1.226 (1.060, 1.421)	0.006	1.192 (1.026, 1.387)	0.023	1.044 (0.891, 1.225)	0.593
No current smoking	1.516 (1.268, 1.818)	<0.001	1.499 (1.246, 1.810)	<0.001	1.386 (1.142, 1.687)	0.001
Regular exercise	0.909 (0.765, 1.078)	0.277	1.067 (0.890, 1.278)	0.480	1.104 (0.919, 1.326)	0.289
Healthy diet	0.669 (0.591, 0.755)	<0.001	0.715 (0.630, 0.811)	<0.001	0.762 (0.669, 0.866)	<0.001
Active cognitive activity	0.610 (0.537, 0.692)	<0.001	0.719 (0.624, 0.827)	<0.001	0.732 (0.634, 0.844)	<0.001

Model 1: unadjusted; Model 2: adjusted for age, sex, residence, marital status, education, and living arrangement; Model 3: further adjusted for drinking alcohol, smoking, regular exercise, cognitive activity, normal weight, diet, hypertension, diabetes, hyperlipidemia, cardiovascular disease, and cerebrovascular disease.

**Table 4:** Association between healthy lifestyle and insomnia risk stratified by smoking status.

Healthy Lifestyle	Model 1		Model 2		Model 3	
	OR (95% CI)	*p*	OR (95% CI)	*p*	OR (95% CI)	*p*
No current smoking						
Unfavorable	ref		ref		ref	
Average	0.842 (0.726, 0.978)	0.024	0.885 (0.760, 1.030)	0.115	0.903 (0.774, 1.052)	0.191
Favorable	0.593 (0.507, 0.694)	<0.001	0.711 (0.599, 0.845)	<0.001	0.738 (0.620, 0.878)	<0.001
*p* for trend	0.769 (0.711, 0.832)	<0.001	0.844 (0.774, 0.920)	<0.001	0.860 (0.788, 0.938)	<0.001
Current smoking						
Unfavorable	ref		ref		ref	
Average	0.721 (0.487, 1.066)	0.102	0.728 (0.486, 1.088)	0.122	0.775 (0.514, 1.167)	0.222
Favorable	0.650 (0.415, 1.010)	0.057	0.649 (0.407, 1.027)	0.067	0.684 (0.424, 1.094)	0.115
*p* for trend	0.799 (0.639, 0.996)	0.047	0.799 (0.634, 1.006)	0.057	0.823 (0.649, 1.041)	0.105

Model 1: unadjusted; Model 2: adjusted for age, sex, residence, marital status, education, and living arrangement; Model 3: adjusted for age, sex, residence, marital status, education, living arrangement, hypertension, diabetes, hyperlipidemia, cardiovascular disease, and cerebrovascular disease.

## Data Availability

The raw data supporting the conclusions of this article will be made available by the authors on request.

## Supplementary Materials

The following supporting information can be downloaded at: https://www.mdpi.com/article/10.3390/clockssleep8020026/s1.

## Abbreviations

The following abbreviations are used in this manuscript:

AIS	Athens Insomnia Scale
BMI	Body Mass Index
CBT-I	Cognitive Behavioral Therapy for Insomnia
CIs	Confidence Intervals
HMACS	Hubei Memory and Aging Cohort Study
OR	Odds Ratios
PSQI	Pittsburgh Sleep Quality Index
SD	Standard Deviation
VIFs	Variance Inflation Factors
