# Supplementary material for: Association of Healthy Lifestyle with Insomnia Among Chinese Older Adults: A Cross-Sectional Study
Source: Clocks Sleep. 2026 May 9;8(2):26. doi: 10.3390/clockssleep8020026 (PMC13214727; doi:10.3390/clockssleep8020026)
Supplement: Supplementary file 1 [file clockssleep-08-00026-s001.zip › clockssleep-4108739-supplementary.pdf]

Association of healthy lifestyle with insomnia among Chinese older adults: a cross-sectional study

Lu Liu, Wen Zhou, Yu Luo, Yueyi Zhang, Dongxi Wang, Ming Chen, Zhiming Wang and Yan Zeng*

Brain Science and Advanced Technology Institute, Wuhan University of Science and Technology, Wuhan 430065, China

Correspondence: Yan Zeng; zengyan68@wust.edu.cn

Figure S1. Proportions of each healthy lifestyle component in general population.
Figure S2. Restricted cubic spline curves showing the nonlinear association between healthy lifestyle score and insomnia
Figure S3. Correlations among individual lifestyle behaviors
Table S1. Baseline Characteristics of Included and Excluded Participants
Table S2. Interaction analyses between individual healthy lifestyle behaviors and insomnia risk.
Table S3. Substitution analysis of healthy lifestyle behaviors and insomnia risk stratified by smoking status
Table S4. Associations between healthy lifestyle and insomnia (insomnia was alternatively defined according to the presence of difficulty initiating sleep, difficulty maintaining sleep, or early-morning awakening with inability to return to sleep)
Table S5. Subgroup analysis stratified by smoking status
Table S6. Association between healthy lifestyle and insomnia risk (n = 2175; AIS ≥ 6)
Table S7. Association between healthy lifestyle and insomnia risk (n = 2754; PSQI > 5)
Table S8. Association between healthy lifestyle and insomnia risk stratified by smoking (never, former or current smoking)
Table S9. Associations between healthy lifestyle and behaviors and insomnia risk using dataset without imputation
Table S10. Associations between healthy lifestyle and insomnia risk stratified by smoking status using dataset without imputation
Table S11. Association between healthy lifestyle score (0–6) and insomnia risk
Table S12. Missing Data Proportion of Covariates
Table S13. Multicollinearity assessment for individual lifestyle behaviors and covariates (VIF values)
Table S14. Multiple correspondence analysis of six healthy lifestyle behaviors: variance explained by each dimension.

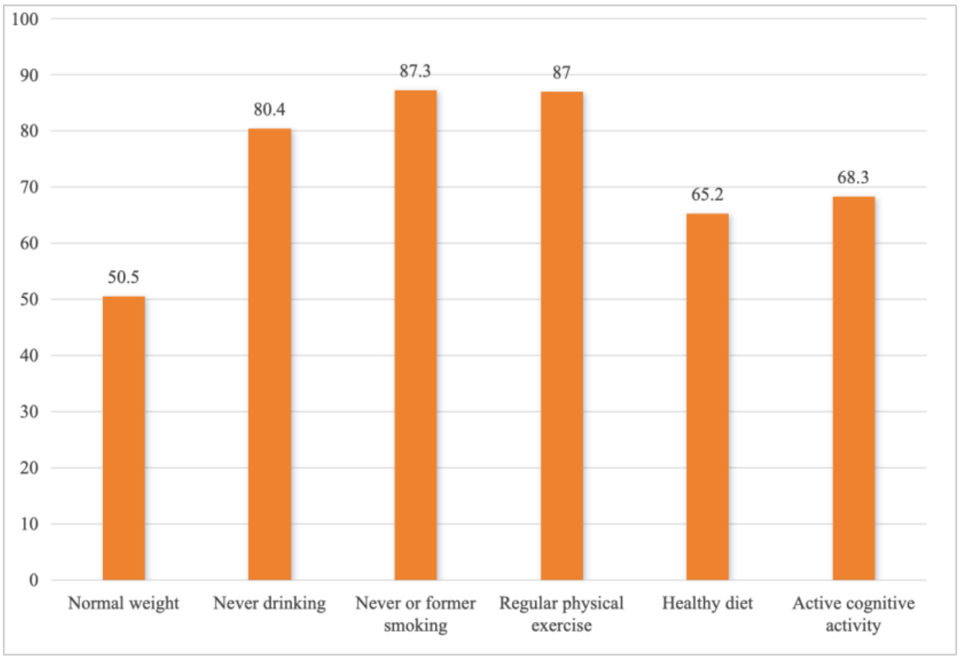

Figure S1. Proportions of each healthy lifestyle component in general population.

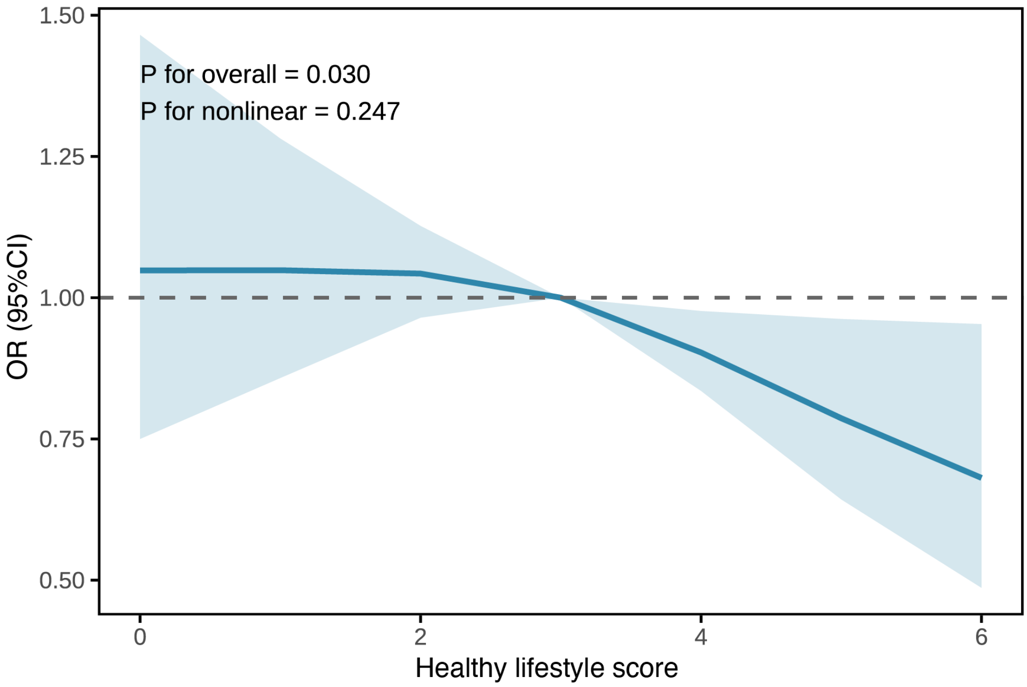

Figure S2. Restricted cubic spline curves showing the nonlinear association between healthy lifestyle score and insomnia**;** Model adjusted for age, sex, residence, marital status, education, living status, BMI, drinking, smoking, healthy diet, regular exercise, cognitive activity, hypertension, diabetes, hyperlipidemia, cardiovascular disease, and cerebrovascular disease.

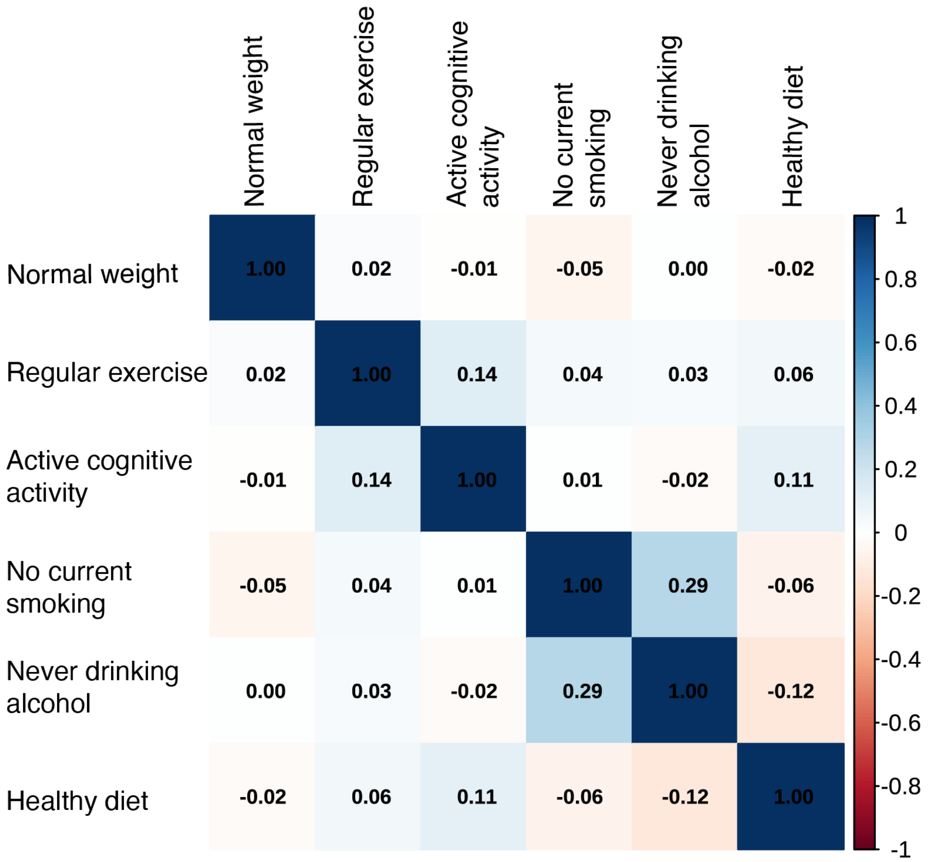

Figure S3. Correlations among individual lifestyle behaviors

Table S1. Baseline Characteristics of Included and Excluded Participants

	Overall (*n* = 12607)	Excluded (*n* = 7678)	Included (*n* = 4929)	*p*	SMD	Missing
Age (mean (SD))	72.06 (6.13)	71.71 (6.41)	72.61 (5.62)	<0.001	0.149	0%
Sex (%)				0.254	0.021	0%
Men	5654 (44.85)	3475 (45.26)	2179 (44.21)			
Women	6953 (55.15)	4203 (54.74)	2750 (55.79)			
Residence (%)				<0.001	0.339	0%
Urban	4629 (36.72)	2331 (30.36)	2298 (46.62)			
Rural	7978 (63.28)	5347 (69.64)	2631 (53.38)			
Marriage (%)				0.058	0.035	2.1%
Married	8712 (70.62)	5227 (69.98)	3485 (71.59)			
Other	3625 (29.38)	2242 (30.02)	1383 (28.41)			
Education (mean (SD))	7.55 (5.21)	8.00 (5.06)	6.85 (5.35)	<0.001	0.222	0.30%
Living arrangement (%)				<0.001	0.237	11.4%
Lives alone	1878 (16.82)	912 (13.33)	966 (22.32)			
With a family member	9290 (83.18)	5929 (86.67)	3361 (77.68)			

Hyperlipidemia (%)				0.295	0.02	2.3%
No	9231 (74.92)	5603 (75.26)	3628 (74.41)			
Yes	3090 (25.08)	1842 (24.74)	1248 (25.59)			
Diabetes (%)				0.759	0.006	1.8%
No	10042 (81.13)	6074 (81.04)	3968 (81.28)			
Yes	2335 (18.87)	1421 (18.96)	914 (18.72)			
Hypertension (%)				0.003	0.055	1.30%
No	5913 (47.54)	3665 (48.62)	2248 (45.87)			
Yes	6526 (52.46)	3873 (51.38)	2653 (54.13)			
Cardiovascular disease (%)				0.031	0.04	2%
No	10190 (82.46)	6220 (83.07)	3970 (81.54)			
Yes	2167 (17.54)	1268 (16.93)	899 (18.46)			
Cerebrovascular disease (%)				<0.001	0.083	2.5%
No	9820 (79.85)	5839 (78.54)	3981 (81.85)			
Yes	2478 (20.15)	1595 (21.46)	883 (18.15)			

Table S2. Interaction analyses between individual healthy lifestyle behaviors and insomnia risk.

Exposure	Main Effect OR (95%CI)	Interaction Term	Interaction OR (95%CI)	*P* for interaction
Healthy diet	0.784 (0.671, 0.915)	active cognitive activity	0.913 (0.696, 1,20)	0.510
Regular exercise	1.43 (0.915, 2.19)	no current smoking	0.731 (0.457, 1.18)	0.195

Table S3. Substitution analysis of healthy lifestyle behaviors and insomnia risk stratified by smoking status

Behavior	Model 1		Model 2		Model 3	
	OR (95%CI)	*p*	OR (95%CI)	*p*	OR (95%CI)	*p*
No current smoking						
Normal weight	0.942 (0.834, 1.065)	0.340	0.948 (0.837, 1.074)	0.402	0.968 (0.853, 1.098)	0.61
Never drinking alcohol	1.115 (0.939, 1.325)	0.214	1.120 (0.939, 1.336)	0.209	1.109 (0.928, 1.324)	0.255
Regular exercise	0.957 (0.798, 1.148)	0.636	1.087 (0.899, 1.316)	0.389	1.101 (0.909, 1.334)	0.325
Healthy diet	0.669 (0.586, 0.763)	<0.001	0.713 (0.623, 0.817)	<0.001	0.743 (0.648, 0.852)	<0.001
Active cognitive activity	0.613 (0.536, 0.702)	<0.001	0.729 (0.628, 0.847)	<0.001	0.729 (0.627, 0.848)	<0.001
Current smoking						
Normal weight	0.891 (0.632, 1.256)	0.509	0.918 (0.647, 1.305)	0.635	0.970 (0.676, 1.391)	0.867
Never drinking alcohol	1.001 (0.711, 1.409)	0.994	0.966 (0.681, 1.371)	0.846	0.986 (0.690, 1.410)	0.94
Regular exercise	1.070 (0.592, 1.931)	0.823	1.080 (0.584, 1.999)	0.805	1.072 (0.572, 2.008)	0.828
Healthy diet	0.770 (0.544, 1.090)	0.140	0.763 (0.534, 1.090)	0.137	0.748 (0.520, 1.075)	0.117

Active cognitive activity	0.675 (0.461, 0.989)	0.044	0.671 (0.441, 1.020)	0.062	0.681 (0.445, 1.042)	0.077

Model 1: unadjusted; Model 2: adjusted for age, sex, residence, marital status, education and living arrangement; Model 3: adjusted for age, sex, residence, marital status, education, living status, BMI, drinking, smoking, healthy diet, regular exercise, cognitive activity, hypertension, diabetes, hyperlipidemia, cardiovascular disease, and cerebrovascular disease.

Table S4. Associations between healthy lifestyle and insomnia (insomnia was alternatively defined according to the presence of difficulty initiating sleep, difficulty maintaining sleep, or early-morning awakening with inability to return to sleep)

	Model 1		Model 2		Model 3	
	OR (95%CI)	*p*	OR (95%CI)	*p*	OR (95%CI)	*p*
Unfavorable	ref		ref		ref	
Average	0.854 (0.714, 1.021)	0.084	0.914 (0.759, 1.101)	0.344	0.934 (0.774, 1.127)	0.474
Average	0.637 (0.527, 0.770)	<0.001	0.800 (0.652, 0.982)	0.033	0.811 (0.659, 0.999)	0.048

Model 1: unadjusted; Model 2: adjusted for age, sex, residence, marital status, education and living arrangement; Model 3: adjusted for age, sex, residence, marital status, education, living status, hypertension, diabetes, hyperlipidemia, cardiovascular disease, and cerebrovascular disease.

Table S5. Subgroup analysis stratified by smoking status

Variable	OR (95%CI)			*P* for interaction
No current smoking				
	Unfavorable	Average	Favorable	
Age_group				0.643
<74years	ref	0.899 (0.743-1.089)	0.699 (0.565-0.865)	
≥75years	ref	0.894 (0.689-1.160)	0.748 (0.551-1.016)	
Residence				0.377
Rural	ref	0.852 (0.669-1.086)	0.666 (0.525-0.845)	
Urban	ref	0.911 (0.744-1.114)	0.794 (0.597-1.053)	
Sex				0.908
Men	ref	0.838 (0.662-1.060)	0.698 (0.536-0.910)	
Women	ref	0.933 (0.760-1.146)	0.733 (0.581-0.924)	
Living arrangement				0.249
Lives alone	ref	0.841 (0.609-1.162)	0.628 (0.422-0.934)	
With family member	ref	0.911 (0.764-1.086)	0.744 (0.612-0.904)	
Years of schooling				0.571
Never	ref	0.839 (0.658-1.068)	0.695 (0.480-1.003)	
≤6years	ref	1.094 (0.761-1.573)	1.016 (0.661-1.557)	
>6years	ref	0.823 (0.644-1.051)	0.641 (0.504-0.815)	
Marital status				0.642
Married	ref	0.896 (0.743-1.082)	0.715 (0.582-0.878)	
Other	ref	0.881 (0.672-1.155)	0.732 (0.526-1.019)	

Hypertension				0.559
No	ref	0.847 (0.664-1.081)	0.659 (0.507-0.857)	
Yes	ref	0.931 (0.762-1.138)	0.754 (0.596-0.953)	
Diabetes				0.186
No	ref	0.889 (0.749-1.056)	0.754 (0.621-0.915)	
Yes	ref	0.919 (0.650-1.301)	0.559 (0.375-0.830)	
Cardiovascular diseases				0.440
No	ref	0.862 (0.725-1.025)	0.691 (0.569-0.839)	
Yes	ref	1.025 (0.731-1.438)	0.821 (0.552-1.222)	
Hyperlipidemia				0.781
No	ref	0.882 (0.737-1.056)	0.719 (0.584-0.884)	
Yes	ref	0.928 (0.686-1.255)	0.703 (0.508-0.973)	
Cerebrovascular disease				0.480
No	ref	0.861 (0.724-1.024)	0.694 (0.572-0.842)	
Yes	ref	1.031 (0.734-1.449)	0.774 (0.513-1.167)	
Current smoking				
Age_group				0.372
<74years	ref	0.821 (0.494-1.366)	0.827 (0.467-1.458)	
≥75years	ref	0.709 (0.327-1.515)	0.442 (0.157-1.142)	
Residence				0.722
Rural	ref	0.576 (0.270-1.222)	0.578 (0.262-1.269)	
Urban	ref	0.909 (0.550-1.501)	0.778 (0.413-1.436)	
Sex				0.658
Men	ref	0.807 (0.424-1.531)	0.713 (0.339-1.476)	
Women	ref	0.707 (0.408-1.222)	0.596 (0.308-1.132)	
Living arrangement				0.949
Lives alone	ref	0.824 (0.377-1.791)	1.209 (0.467-3.108)	
With family member	ref	0.727 (0.443-1.191)	0.601 (0.340-1.052)	
Years of schooling				0.858
Never	ref	0.663 (0.228-1.862)	0.638 (0.179-2.078)	
≤6years	ref	1.075 (0.528-2.192)	0.783 (0.327-1.821)	
>6years	ref	0.628 (0.336-1.172)	0.564 (0.282-1.117)	
Marital status				0.266
Married	ref	0.695 (0.426-1.131)	0.512 (0.289-0.897)	
Other	ref	0.812 (0.364-1.792)	1.444 (0.573-3.624)	
Hypertension				0.488
No	ref	0.785 (0.442-1.388)	0.866 (0.441-1.681)	
Yes	ref	0.781 (0.420-1.453)	0.567 (0.274-1.149)	
Diabetes				0.197
No	ref	0.781 (0.500-1.217)	0.697 (0.416-1.158)	
Yes	ref	0.716 (0.185-2.796)	0.790 (0.177-3.483)	
Cardiovascular				0.250

diseases				
No	ref	0.963 (0.608-1.529)	0.677 (0.388-1.168)	
Yes	ref	0.257 (0.082-0.743)	0.584 (0.194-1.702)	
Hyperlipidemia				0.861
No	ref	0.857 (0.540-1.361)	0.718 (0.423-1.209)	
Yes	ref	0.660 (0.226-1.873)	0.520 (0.138-1.819)	
Cerebrovascular disease				0.298
No	ref	0.945 (0.588-1.522)	0.830 (0.482-1.422)	
Yes	ref	0.608 (0.237-1.523)	0.380 (0.111-1.182)	

Model adjusted for age, sex, residence, marital status, education, living status, BMI, drinking, smoking, healthy diet, regular exercise, cognitive activity, hypertension, diabetes, hyperlipidemia, cardiovascular disease, and cerebrovascular disease.

Table S6. Association between healthy lifestyle and insomnia risk (n=2175; AIS ≥6)

Healthy lifestyle	Model 1		Model 2		Model 3	
	OR (95%CI)	*p*	OR (95%CI)	*p*	OR (95%CI)	*p*
Unfavorable	ref		ref		ref	
Average	0.812 (0.658, 1.001)	0.051	0.858 (0.690, 1.065)	0.165	0.887 (0.712, 1.104)	0.283
Favorable	0.635 (0.504, 0.798)	<0.001	0.900 (0.690, 1.171)	0.432	0.940 (0.719, 1.228)	0.648
P for trend	0.798 (0.712, 0.894)	0.001	0.937 (0.823, 1.068)	0.333	0.960 (0.841, 1.096)	0.543

Model 1: unadjusted; Model 2: adjusted for age, sex, residence, marital status, education, and living arrangement; Model 3: adjusted for age, sex, residence, marital status, education, living arrangement, hypertension, diabetes, hyperlipidemia, cardiovascular disease, and cerebrovascular disease.

Table S7. Association between healthy lifestyle and insomnia risk (n=2754; PSQI >5)

Healthy lifestyle	Model 1		Model 2		Model 3	
	OR (95%CI)	*p*	OR (95%CI)	*p*	OR (95%CI)	*p*
Unfavorable	ref		ref		ref	
Average	1.031 (0.862, 1.233)	0.740	1.044 (0.869, 1.255)	0.643	1.042 (0.866, 1.255)	0.660
Favorable	0.714 (0.590, 0.863)	0.001	0.796 (0.650, 0.975)	0.027	0.808 (0.658, 0.991)	0.041
P for trend	0.849 (0.772, 0.933)	0.001	0.897 (0.811, 0.992)	0.034	0.904 (0.816, 1.001)	0.052

Model 1: unadjusted; Model 2: adjusted for age, sex, residence, marital status, education, and living arrangement; Model 3: adjusted for age, sex, residence, marital status, education, living arrangement, hypertension, diabetes, hyperlipidemia, cardiovascular disease, and cerebrovascular disease.

Table S8. Association between healthy lifestyle and insomnia risk stratified by smoking (never, former or current smoking)

Healthy lifestyle	Model 1		Model 2		Model 3	
	OR (95%CI)	*p*	OR (95%CI)	*p*	OR (95%CI)	*p*

Never smoking (n = 3819)						
Unfavorable	ref		ref		ref	
Average	0.795 (0.677, 0.933)	0.005	0.849 (0.720, 1.001)	0.051	0.860 (0.728, 1.015)	0.074
Favorable	0.561 (0.474, 0.664)	<0.001	0.690 (0.573, 0.831)	<0.001	0.708 (0.587, 0.854)	<0.001
Former smoking (n = 488)						
Unfavorable	ref		ref		ref	
Average	1.006 (0.656, 1.540)	0.978	1.045 (0.677, 1.609)	0.843	1.131 (0.725, 1.762)	0.587
Favorable	0.662 (0.402, 1.074)	0.099	0.713 (0.426, 1.179)	0.191	0.767 (0.449, 1.298)	0.327
Current smoking (n = 622)						
Unfavorable	ref		ref		ref	
Average	0.721 (0.487, 1.066)	0.102	0.728 (0.486, 1.088)	0.122	0.775 (0.514, 1.167)	0.222
Favorable	0.650 (0.415, 1.010)	0.057	0.649 (0.407, 1.027)	0.067	0.684 (0.424, 1.094)	0.115

Model 1: unadjusted; Model 2: adjusted for age, sex, residence, marital status, education, and living arrangement; Model 3: adjusted for age, sex, residence, marital status, education, living arrangement, hypertension, diabetes, hyperlipidemia, cardiovascular disease, and cerebrovascular disease.

Table S9. Associations between healthy lifestyle and behaviors and insomnia risk using dataset without imputation

Healthy lifestyle	Model 1		Model 2		Model 3	
	OR (95%CI)	p	OR (95%CI)	p	OR (95%CI)	p
Unfavorable	ref		ref		ref	
Average	0.939 (0.820, 1.075)	0.360	0.978 (0.847, 1.129)	0.760	0.989 (0.855, 1.145)	0.883
Favorable	0.690 (0.596, 0.798)	<0.001	0.819 (0.691, 0.970)	0.021	0.842 (0.709, 1.001)	0.051
P for trend	0.835 (0.777, 0.897)	<0.001	0.912 (0.838, 0.991)	0.030	0.924 (0.849, 1.006)	0.070
Behaviors						
Normal weight	0.925 (0.825, 1.036)	0.179	0.932 (0.824, 1.054)	0.259	0.945 (0.832, 1.073)	0.382
Never drinking alcohol	1.226 (1.060, 1.421)	0.006	1.217 (1.040, 1.425)	0.015	1.087 (0.920, 1.285)	0.329
Regular exercise	1.516 (1.268, 1.818)	<0.001	1.499 (1.237, 1.822)	<0.001	1.392 (1.137, 1.709)	0.001
Healthy diet	0.909 (0.765, 1.078)	0.277	1.005 (0.824, 1.225)	0.957	1.046 (0.853, 1.280)	0.665
Active cognitive activity	0.669 (0.591, 0.755)	<0.001	0.743 (0.650, 0.849)	<0.001	0.790 (0.688, 0.906)	<0.001

Model 1: unadjusted; Model 2: adjusted for age, sex, residence, marital status, education and living arrangement; Model 3: adjusted for age, sex, residence, marital status, education, living arrangement, hypertension, diabetes, hyperlipidemia, cardiovascular disease, and cerebrovascular disease. In the analysis of each healthy lifestyle behavior, the reference category was the unhealthy or non-participation group. Specifically, abnormal weight was used as the reference for normal weight; current drinking alcohol for never drinking; current smoking for no current smoking; physical inactivity for regular exercise; unhealthy diet for healthy diet; and inactive cognitive activity for active cognitive activity.

Table S10. Associations between healthy lifestyle and insomnia risk stratified by smoking status using dataset without imputation

Healthy lifestyle	Model 1		Model 2		Model 3	
	OR (95%CI)	*p*	OR (95%CI)	*p*	OR (95%CI)	*p*
No current smoking						
Unfavorable	ref		ref		ref	
Average	0.842 (0.726, 0.978)	0.024	0.892 (0.762, 1.045)	0.157	0.907 (0.772, 1.066)	0.235
Favorable	0.593 (0.507, 0.694)	<0.001	0.724 (0.603, 0.869)	<0.001	0.744 (0.617, 0.896)	0.002
P for trend	0.769 (0.711, 0.832)	<0.001	0.852 (0.778, 0.934)	<0.001	0.864 (0.788, 0.949)	0.002
Current smoking						
Unfavorable	ref		ref		ref	
Average	0.650 (0.415, 1.010)	0.057	0.777 (0.480, 1.246)	0.298	0.807 (0.491, 1.319)	0.396
Favorable	0.799 (0.639, 0.996)	0.047	0.869 (0.684, 1.102)	0.248	0.891 (0.695, 1.139)	0.358
P for trend	0.721 (0.487, 1.066)	0.102	0.749 (0.493, 1.134)	0.172	0.807 (0.526, 1.237)	0.324

Model 1: unadjusted; Model 2: adjusted for age, sex, residence, marital status, education and living arrangement; Model 3: adjusted for age, sex, residence, marital status, education, living arrangement, BMI, drinking, smoking, healthy diet, regular exercise, cognitive activity, hypertension, diabetes, hyperlipidemia, cardiovascular disease, and cerebrovascular disease.

Table S11. Association between healthy lifestyle score (0-6) and insomnia risk

	Model 1		Model 2		Model 3	
	OR (95%CI)	*p*	OR (95%CI)	*p*	OR (95%CI)	*p*
Healthy lifestyle score	0.881 (0.835, 0.929)	<0.001	0.930 (0.877, 0.986)	0.015	0.941 (0.887, 0.998)	0.043

Model 1: unadjusted; Model 2: adjusted for age, sex, residence, marital status, education and living arrangement; Model 3: adjusted for age, sex, residence, marital status, education, living arrangement, BMI, drinking, smoking, healthy diet, regular exercise, cognitive activity, hypertension, diabetes, hyperlipidemia, cardiovascular diseases, and cerebrovascular diseases.

Table S12. Missing Data Proportion of Covariates

Variables	Missing proportion
Age	0%
Sex	0%
Education	0%
Residence	0%
Marriage	1.2%
Living arrangement	12.2%
Hypertension	0.6%
Diabetes	0.9%
Hyperlipidemia	1.1%

Cardiovascular disease	1.2%
Cerebrovascular disease	1.3%

Table S13. Multicollinearity assessment for individual lifestyle behaviors and covariates (VIF values)

Variables	VIF
Normal weight	1.030
No current smoking	1.343
Never drinking alcohol	1.304
Regular exercise	1.092
Healthy diet	1.056
Active cognitive activity	1.218
Age	1.121
Sex	1.024
Education	2.553
Residence	2.595
Marriage	1.761
Living arrangement	1.651
Hypertension	1.099
Diabetes	1.067
Hyperlipidemia	1.147
Cardiovascular disease	1.085
Cerebrovascular disease	1.063

Table S14. Multiple correspondence analysis of six healthy lifestyle behaviors: variance explained by each dimension.

Dimension	Variance explained (%)	Cumulative variance (%)
Dim1	22.49	22.49
Dim2	19.97	42.46
Dim3	17.01	59.47
Dim4	14.70	74.17
Dim5	14.14	88.31
Dim6	11.69	100.00

Multiple correspondence analysis (MCA) was conducted to explore potential clustering patterns among the six lifestyle behaviors. The first six dimensions explained 100% of the variance, with Dim1 accounting for 22.49%, Dim2 19.97%, Dim3 17.01%, Dim4 14.70%, Dim5 14.14%, and Dim6 11.69% of the variance, indicating no dominant clustering pattern.
